# Identification of Small‐Molecule Inhibitors of the Antiapoptotic Protein Myeloid Cell Leukaemia‐1 (Mcl‐1)

**DOI:** 10.1002/cmdc.201500488

**Published:** 2015-11-30

**Authors:** Andrew M. Beekman, Maria A. O'Connell, Lesley A. Howell

**Affiliations:** ^1^ School of PharmacyUniversity of East AngliaNorwich Research ParkNorwichNorfolkNR4 7TJUK

**Keywords:** Bcl-2, cancer, Mcl-1, molecular modelling, protein–protein interactions

## Abstract

Protein–protein interactions (PPIs) control many cellular processes in cancer and tumour growth. Of significant interest is the role PPIs play in regulating apoptosis. The overexpression of the antiapoptosis regulating Bcl‐2 family of proteins is commonly observed in several cancers, leading to resistance towards both radiation and chemotherapies. From this family, myeloid cell leukemia‐1 (Mcl‐1) has proven the most difficult to target, and one of the leading causes of treatment resistance. Exploiting the selective PPI between the apoptosis‐regulating protein Noxa and Mcl‐1, utilising a fluorescence polarization assay, we have identified four small molecules with the ability to modulate Mcl‐1. The identified compounds were computationally modelled and docked against the Mcl‐1 binding interface to obtain structural information about their binding sites allowing for future analogue design. When examined for their activity towards pancreatic cell lines that overexpress Mcl‐1 (MiaPaCa‐2 and BxPC‐3), the identified compounds demonstrated growth inhibition, suggesting effective Mcl‐1 modulation.

Protein–protein interactions (PPIs) are involved in many cellular processes^1^ and have therefore emerged as an attractive drug target in recent years. Specifically they have been shown to regulate several processes within cancer and tumour growth,^2^ and targeting PPIs is considered a promising strategy towards next generation anticancer therapeutics.^3^ However, PPIs pose a considerable challenge to the medicinal chemistry community due to their large, flat, shallow interfaces, which possess a high degree of flexibility and thus are deemed problematic for drug design.^4^ Despite this, small molecules have proved successful at modifying their actions.^5^ In particular, modulation of the p53–MDM2 interaction and Bcl‐2 family interactions has been achieved by drug candidates in clinical trials, overcoming the perception that PPIs are “undruggable”.^6^

The PPIs of the Bcl‐2 family play an important role in apoptosis as key regulators, a process that is highly conserved and controlled.^7^ The family consists of both pro‐ and anti‐apoptotic proteins, and there is a careful balance within a cell that controls its fate.2a It is believed that upon receipt of cellular stress, the proapoptotic proteins BAX and BAK are activated by the BH3‐only proteins, where they migrate to the surface of the mitochondria. Here, they form oligomers and insert themselves into the outer mitochondrial membrane forming pores This process is known as mitochondrial outer membrane permeabilisation (MOMP). This in turn leads to the rapid and irreversible release of cytochrome c from the mitochondria into the cytosol, which activates downstream caspases resulting in apoptosis. High levels of the antiapoptotic proteins (e.g., Bcl‐2, Bcl‐xL and Mcl‐1) are often observed in cancer, and they not only contribute to the development of the tumour but also confer resistance to current therapies including chemotherapy and radiation treatment.2a In particular, overexpression of myeloid cell leukemia‐1 (Mcl‐1) is one of the most common forms of genetic abnormalities in cancer,^8^ with a variety of human cancers, including pancreatic cancer, exhibiting high levels of the protein.^9^

The antiapoptotic Bcl‐2 family of proteins are well‐validated anticancer targets. The most successful small‐molecule inhibitors to date, ABT‐737 and its orally available analogue ABT‐263 (Navitoclax), inhibit Bcl‐2 and Bcl‐xL with sub‐nanomolar affinity.^10^ Although ABT‐263 has entered clinical trials, like most small‐molecule Bcl‐2 inhibitors, it does not inhibit Mcl‐1 and lacks efficacy in tumours with high levels of Mcl‐1 rendering it ineffective as a single agent.^11^ Furthermore, Mcl‐1 overexpression has been linked to resistance observed against paclitaxel and vincristine,^12^ as well as the first‐line treatment for pancreatic cancer, gemcitabine.^9^ Therefore, compounds that specifically target Mcl‐1 have the potential to overcome this resistance. The first selective Mcl‐1 inhibitor was identified as recently as 2010;^13^ however, there are currently no compounds undergoing clinical trials that target Mcl‐1.

Pancreatic ductal adenocarcinoma has the lowest survival rates of any cancer.^14^ According to Cancer Research UK, less than 4 % of patients diagnosed with the disease will survive for at least five years, and this drops to less than 3 % over a ten‐year period.^15^ More worrying though is the fact that these figures have not changed over the last 40 years despite the research efforts of many groups. Recent studies have shown that downregulating Mcl‐1 enhances the sensitivity of human pancreatic cancer cells to gemcitabine and radiation, resulting in increased levels of apoptosis.^9^, ^16^ Furthermore, knockdown of Mcl‐1 in pancreatic cancer cells treated with ABT‐737 triggers apoptosis, indicating Mcl‐1 as an important and significant therapeutic target in this type of cancer.^17^

The Bcl‐2 family has proteins that regulate the activity of Bcl‐2, Bcl‐xL and Mcl‐1, including Bim, Bid, Puma, Bad, and Noxa. Of the apoptosis regulator proteins, Noxa displays the greatest selectivity towards Mcl‐1, binding exclusively to Mcl‐1 and Bfl‐1/A1.^18^ Interestingly, to the best of our knowledge, only one other group has exploited this selectivity to explore small‐molecule Mcl‐1 binding.^19^ Herein, we report the exploration of the Mcl‐1 binding pocket and the identification of novel leads for Mcl‐1 inhibition utilising the binding domain of the selective apoptosis regulating protein Noxa.

To identify potential inhibitors of Mcl‐1, we employed a competitive fluorescence polarization (FP) assay similar to the one we reported for identifying inhibitors of the p53–Mdm2 interaction.^20^ The assay utilises the 19‐residue alpha helix binding domain of NoxaB (AAQLRRIGDKVNLRQKLLN) tagged on the N terminus with fluorescein isothiocyanate (FITC) and measures the competitive binding by the displacement of the tagged peptide from Mcl‐1, resulting in an increase in fluorescence polarisation. The NoxaB peptide AAQLRRIGDKVNLRQKLLN was synthesised on Rink amide resin to generate the amide at the C terminus. Fluorenylmethyloxycarbonyl (Fmoc)‐aminohexanoic acid was subsequently coupled to the N terminus followed by coupling with FITC to generate the fluorescently tagged NoxaB peptide (FITC‐NoxaB). A chimeric mouse/human Mcl‐1 protein, previously reported by Colman^21^ and used in an FP assay reported by Yu and Wang,^22^ was employed by us in the polarization assay. The chimeric Mcl‐1 protein has good solubility in water and maintains the biological function of human Mcl‐1, with the BH3 binding groove consisting entirely of the human Mcl‐1 sequence. An acetylated NoxaB peptide without the aminohexanoic acid or FITC tag was used as a positive control and exhibited an IC_50_ value of 0.65 μm and a *K*_i_ value of 0.22 μm. To confirm the reproducibility of our data, a Z‐prime test was undertaken producing a result of 0.78 indicating the assay is suitable for high‐throughput screening.

Following optimisation of the FP assay, we screened the US National Cancer Institute (NCI) diversity set IV for potential Mcl‐1 inhibitors. The NCI diversity set is a collection of 1600 natural and synthetic compounds with a diverse structural landscape that have been evaluated as potential anticancer agents. Compounds were screened initially at a concentration of 100 μm, and seven compounds were identified as potential hits (0.44 % hit rate). A full dose–response assay revealed that four of these compounds (Figure 1) displayed an IC_50_ value of less than 20 μm in subsequent dose–response assays (Table 1).

**Figure 1:**
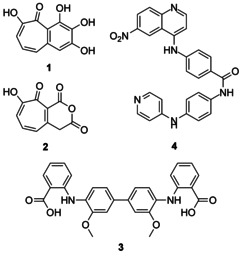
Structures of US National Cancer Institute (NCI) diversity set IV Mcl‐1/mNoxa binding inhibitors **1**–**4**.

**Table 1:** Activity of compounds **1**–**4** against Bcl‐2 family proteins including binding inhibition (IC_50_) of FITC‐mNoxa to Mcl‐1, binding affinity constant (*K*_i_), and cell growth inhibition (IC_50_) of pancreatic cancer cells lines MiaPaCa‐2 and BxPC‐3.

Compd	Mcl‐1^[a]^		MiaPaCa‐2	BxPC‐3
	FP IC_50_ [μm]	FP *K*_i_ [μm]	IC_50_ [μm]^[b]^	IC_50_ [μm]^[b]^
**1**	1.57±0.97	0.80±0.30	17.66±9.34	>100
**2**	13.70±7.35	6.99±2.40	>100	>100
**3**	5.78±0.97	2.95±0.49	>100	>100
**4**	2.14±2.94	1.09±1.50	88.82±7.35	15.11±10.95

Data represent the mean±SD of *n*=3 independent experiments performed in [a] duplicate or [b] triplicate.

The structure of purpurogallin (**1**) first appears in the literature in 1882 and is obtained by the oxidation of pyrogallol.^23^ The potential of purpurogallin as a Bcl‐2 family binder has been recognised since 2003,^24^ and in 2011, it was part of a patent that covers small molecules that modulate Mcl‐1.^25^ Compound **1** displayed sub‐micromolar binding affinity (*K*_i_=0.80 μm) towards Mcl‐1. Our findings support those already reported in the literature and demonstrate the ability of purpurogallin to bind competitively to Mcl‐1. The structure of purpurogallin derivative **2** has, to the best of our knowledge, only appeared in the literature twice previously,^26^ and here, we have identified it as a Mcl‐1 inhibitor with micromolar affinity (*K*_i_=6.99 μm). The decreased binding affinity, when compared with compound **1**, suggests that the triphenol moiety is of importance for binding.

Redoxal (**3**) was first reported in 1960 as a redox indicator in alkaline media.^27^ It is part of a patent from Cadone and co‐workers that details biphenazine compounds for treating hematopoietic cancers.^28^ Compound **3** possessed a binding affinity in the low micromolar region (*K*_i_=2.95 μm). This could suggest that the activity observed in hematopoietic cancers by Cardone could be a result of Mcl‐1 modulation, as it has been widely reported that hematopoietic cancers cells survive for extended periods due to Bcl‐2 family overexpression.^29^ Compound **4** was first prepared by Cain and Atwell in 1972 as a potential antitumour agent but was shown to be inactive in the lymphocytic leukaemia cell line L1210.^30^ It has been identified as a modulator of protein–RNA interactions, specifically the Gag polyprotein and the viral RNA packaging signal.^31^ Here, we have identified **4** as a novel Mcl‐1 binder with low micromolar affinity (*K*_i_=1.09 μm). Of the identified compounds, only compound **1** approached the potency of the untagged NoxaB control (*K*_i_=0.22 μm), but all compounds demonstrated sufficient activity to be worthy of consideration for further elaboration.

In order to rationalise the binding activity of **1**–**4** and to facilitate the structure‐based design of analogues, computational docking experiments were performed. Computations were based on the published structure of mouse Mcl‐1 bound to a modified Noxa BH3 peptide (PDB ID: 2NLA).^21^ The modified Noxa and Mcl‐1 make key interactions at the Mcl‐1 amino acids Met 212, Lys 215, Asn 223, Asp 256 and Arg 263 (Figure 2 a).^21^ Compound **1** was predicted to bind in a groove created by the BH1 and BH3 domains, displaying electrostatic interactions with Arg 222 and Val 321 (Figure 2 b). It sits in a hydrophobic pocket that is also occupied by the mNoxa peptide. Compound **2** appears to bind in a similar pocket to **1**, shifted slightly to form electrostatic interactions with Asn 223 (comparable to mNoxa) and His 224 (Figure 2 c). The docking of compound **3** indicated that it might act as a BH3 mimetic, binding in the mNoxa binding groove created by the BH1, BH2 and BH3 domains. Electrostatic interactions with His 224 and Asn 260 were observed. The ligand is predicted to bind across the hydrophobic groove, and aromatic stacking interactions are potentially observed between **3** and Phe 319 (Figure 2 d). Similarly, the results of the docking of **4** suggest that it too may be a BH3 mimetic, binding in a similar manner to compound **3**. Docking suggested electrostatic interactions to Arg 222 and the backbone of Gly 219 (Figure 2 e).

**Figure 2:**
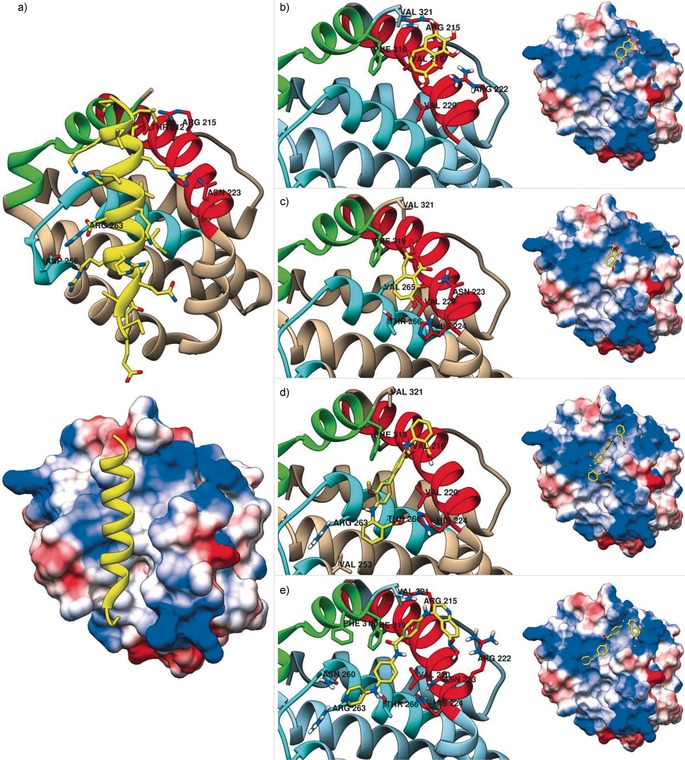
Computational docking of mNoxa and compounds **1**–**4** to Mcl‐1 (PDB ID: 2NLA 21). a) Reproduction of the 2NLA crystal structure displaying mNoxa binding to Mcl‐1 in the largely hydrophobic BH3 domain binding groove create by the BH1, BH2 and BH3 domains of Mcl‐1, and docking results showing b) **1** in a largely hydrophobic pocket, c) **2** in a hydrophobic pocket, d) **3** in a similar groove to mNoxa, and e) **4** in a similar groove to mNoxa. Left: ribbon representation of Mcl‐1 (BH1 (blue), BH2 (green) and BH3 (red) domains) displaying residue interactions of interest (ligand: yellow; atom colours: H=white, N=blue, O=red); Right: the columbic surface representation showing electron rich areas (blue), electron poor areas (red), and hydrophobic areas (white).

The diverse range of binding interactions predicted by molecular docking suggests that improvements could be made to all compounds. A combination of the binders could be explored, a technique that has been shown to be highly successful at targeting protein–protein interactions.^11^, ^32^ Initial considerations would explore the incorporation of the triphenol moiety of **1**, which appears to play an important role in binding, into compound **4**. Additionally, the alteration of aromatic groups in **4**, utilising palladium cross coupling chemistry during synthesis, could be exploited to increase interactions with Phe 319, an interaction observed in the binding of **1** and **3**. The studies also suggest that potential ligands could perform well as selective Mcl‐1 binders even if they do not mimic the BH3 domain, and that hydrophobic binding may prove to be more effective than electrostatic interactions.

Finally, we examined compounds **1**–**4** for their ability to inhibit the growth and induce cell death in pancreatic cell lines MiaPaCa‐2 and BxPC‐3. Both cell lines show an increased expression in Mcl‐1 and Bcl‐xL, but only MiaPaCa‐2 shows an increase in Bcl‐2.^33^ A summary of the cell growth inhibition is presented in Table 1. Compounds **2** and **3** displayed no significant activity towards either of the cell lines, with IC_50_ values greater than 100 μm. Compound **1** inhibited the growth of MiaPaCa‐2 with an IC_50_ value of 17.6 μm, but displayed inhibition greater than 100 μm towards BxPC‐3. Compound **4** demonstrated inhibition in both MiaPaCa‐2 and BxPC‐3, with IC_50_ values of 88.8 μm and 15.1 μm, respectively. These results could indicate that **1** is capable of modulating Bcl‐2 in pancreatic cancer, but is ineffective towards Mcl‐1. Compound **4** shows micromolar inhibition in both cell lines, perhaps demonstrating an ability to effectively modulate Bcl‐2 family interactions. In fact, Takahashi and co‐workers have recently reported that Bcl‐xL and Mcl‐1 might co‐operatively play a role in the apoptotic cell death of pancreatic cancer and that targeting both proteins may be a viable therapeutic strategy.^33^

In summary, we have demonstrated that the selective Mcl‐1 ligand Noxa can be exploited to identify regulators of the antiapoptotic protein Mcl‐1. The modified Noxa peptide was utilised in a fluorescence polarization assay to screen 1600 compounds, identifying four hits with binding affinities of less than 10 μm. These compounds were used to explore the binding pocket of Mcl‐1 computationally, allowing for the binding of mNoxa to be compared with the identified hits (compounds **1**–**4**), identifying potential synthetic enhancements for the novel binders **2** and **4**. The viability of cells treated with compounds **1**–**4** was examined, demonstrating the ability of **1** and **4** to inhibit the growth of pancreatic cell lines that overexpress Mcl‐1 (MiaPaCa‐2 and BxPC‐3). Examination of target selectivity, structure–activity relationship examination, and mode of cell growth inhibition of compounds **1**–**4** will be reported in due course.

## Supporting information

As a service to our authors and readers, this journal provides supporting information supplied by the authors. Such materials are peer reviewed and may be re‐organized for online delivery, but are not copy‐edited or typeset. Technical support issues arising from supporting information (other than missing files) should be addressed to the authors.

Supplementary
