# Supplementary material for: Identification of Small‐Molecule Inhibitors of the Antiapoptotic Protein Myeloid Cell Leukaemia‐1 (Mcl‐1)
Source: ChemMedChem. 2015 Nov 30;11(8):840–4. doi: 10.1002/cmdc.201500488 (PMC4848766; doi:10.1002/cmdc.201500488)
Supplement: Supplementary file 1 — Supplementary [file CMDC-11-840-s001.pdf]

CHEMMEDCHEM Chemistry & Drug Discovery

Supporting Information

Identification of Small-Molecule Inhibitors of the Antiapoptotic Protein Myeloid Cell Leukaemia-1 (Mcl-1)

Andrew M. Beekman, Maria A. O'Connell, and Lesley A. Howell*[a]

cmdc_201500488_sm_miscellaneous_information.pdf

Supplementary Information

Contents

1. General Procedures……………………………………………………………………………………………….2

2. Protein Expression………………………………………………………………………………………………..2

3. Peptide Synthesis…………………………………………………………………………………………………..2

4. Fluorescence Polarization Binding Assay……………………………………………………………..3

5. Molecular Docking………………………………………………………………………………………………...4

6. Cell Culture………4

7. MTS Cytotoxicity Assay…………………………………………………………………………………………..4

8. UV Absorbance Screen……………………………………………………………………………………………4

1. General Procedures

Reagents and Solvents

All chemicals were reagent grade and were purchased from Sigma Aldrich, Fisher Scientific and Tokyo Chemical Industry. Fmoc-amino acids and coupling reagents were purchased from Novabiochem or AGTC Bioproducts. Anhydrous solvents were bought from Sigma Aldrich and assumed to conform to specification.

Physical Characterisation and Spectroscopic Techniques

MALDI was performed on Kratos Analytical Axima MALDI-TOF. The matrix, α-cyano-4-hydroxycinnamic acid (>98% Sigma), was prepared in ethanol at a concentration of 10 mg/mL. Compounds to be analysed were prepared at a concentration of 1 mg/mL in methanol. Samples were prepared by mixing equal volumes (5 µL) of matrix and compound. The MALDI experiment was performed in reflectron mode under positive ionisation conditions. The following parameters were used: 1000 profiles per sample, shots off, ion gate off, pulsed extraction on, max laser rep rate 10.

Chromatographic Techniques

Automatic flash chromatography was performed on a Biotage Isolera Four, using Biotage SNAP Cartridges KP-C18-HS. Solvent A = 95% Water + 5% Methanol + 0.05% TFA and Solvent B = 95% MeOH + 5% Water + 0.05% TFA, gradient from 0 → 100% B over 60 minutes. Detection wavelength 214nm.

Analytical RP-HPLC was performed on an Agilent 1200 using an Agilent eclipse XDB-C18 column, 4.6 x 150mm, 5µM and a flow rate of 1 mL/min. Solvent A = Water + 0.05% TFA and Solvent B = MeOH + 0.05% TFA. Gradient 5% B → 95% B over 20 minutes. Detection wavelength 214 nm.

2. Protein Expression

The Mcl-1 protein used in this study is the same construction as the Mcl-1 described by Yu and Wang,[1] amino acid residues 152-189 of mouse Mcl-1 and amino acid residues 209-327 of human Mcl-1. The plasmid was generously provided by Yu and Wang. The protein with an N-terminal 8 x His tag was expressed in *E. coli* BL21(DE3)pLysS cells. Cells were grown at 37 °C in LB medium containing 100 µg/mL ampicillin and 34 µg/mL chloramphenicol to an OD_{600} value at 0.6. Protein expression was induced by 0.4 mM IPTG at 30 °C for 4 h. Cells were lysed in 25 mM Tris-HCl, pH 8.0 buffer containing 300 mM NaCl, 5 mM βME and 0.1 mg/mL PMSF. His-TEV-Mcl-1 protein was purified from the soluble fraction using Ni-NTA resin (QIAGEN), following the manufacturer's instructions.

3. Peptide Synthesis

FITC-NoxaB Peptide Synthesis

The nineteen amino acid mouse NoxaB peptide AAQLRRIGDKVNLRQKLLN was synthesised on Rink amide resin (resin loading 0.6 mmol/g) using an automated peptide synthesiser. 100 mg of Rink Amide MBHA resin (0.06 mmol) was suspended in DMF (2 mL) and allowed to swell for 20 minutes. The DMF was drained from the peptide vessel and Fmoc deprotection was carried out by addition of 2 x 2 mL of 40% piperidine in DMF, which was vortexed for 10 minutes. This was removed and the resin was washed with DMF (3 x 2 mL). The resin was then treated with a solution of Fmoc-Asn-Trt (4 equiv. compared to resin loading), to which HBTU (3.9 equiv.) and HOBt (4 equiv.) and DIPEA (8 equiv.) in DMF were added. The mixture was then vortexed for 30 min. The vessel was drained and the resin washed with DMF (3 x 2 mL). The coupling reaction was then repeated followed by Fmoc deprotection (2 x 2 mL 40% piperidine in DMF, 10 min) and finally the resin was washed with DMF. Subsequent

amino acids were coupled in an identical fashion. After the final amino acid coupling reaction (alanine) and Fmoc deprotection, the resin was treated with Fmoc-aminohexanoic acid (Ahx, 4 equiv.), HBTU (3.9 equiv.), HOBt (4 equiv.) and DIPEA (8 equiv.) and reacted for 45 minutes. Subsequent Fmoc deprotection was followed by coupling with FITC (1.5 equiv.) and DIPEA (2 equiv.) in DMF overnight. The resin was washed with DCM (x 3) and 1:1 MeOH:DCM (x 3) to remove any residual DMF. The peptide was cleaved from the resin using 95:2.5:2.5 TFA:TIPS:H_2O (5 mL) and shaken for 3 h after which the cleavage cocktail was drained into a round bottom flask. The resin was washed with TFA (x 3) and the solutions combined and concentrated in vacuo. The peptide was precipitated using cold diethyl ether and filtered. The peptide was purified using automated reversed phase flash chromatography and lyophilised from water to yield a yellow solid. This was subsequently analysed using RP-HPLC (RT = 13.5 min) and MALDI (m/z 2707.34 M+H observed).

Synthesis of NoxaB peptide

The NoxaB peptide AAQLRRIGDKVNLRQKLLN synthesis was performed as above but the peptide was acetylated at the N-terminus after the addition of the final amino acid (alanine) using acetyl chloride (4 equiv.) and DIPEA (4 equiv.) in DMF and shaken for 45 minutes. The resin was washed with DMF (x 3), DCM (x 3) and 1:1 MeOH:DCM (x 3) to remove any residual DMF. The peptide was cleaved from the resin using 95:2.5:2.5 TFA:TIPS:H_2O (5 mL) and shaken for 3 h after which the cleavage cocktail was drained into a round bottom flask. The resin was washed with TFA (x 3) and the solutions combined and concentrated in vacuo. The peptide was precipitated using cold diethyl ether and filtered. The peptide was purified using automated reversed phase flash chromatography and lyophilised from water to yield a yellow solid. This was subsequently analysed using RP-HPLC (RT = 12.8 min) and MALDI (m/z 2248.85 M+H observed).

4. Fluorescence Polarization Binding Assay

Fluorescence polarisation was carried out on a BMG Labtech clariostar microplate reader with a fluorescence polarisation optic measuring at 482/530 nm. Low-binding, Corning 96-well black plates were used and all reagents used in the assay were biological grade and purchased from Sigma Aldrich and Novabiochem. All solutions were made using MilliQ water

For binding each well contained 10 µL of Mcl-1 protein in 10-fold dilutions (0.37 pM to 3.7 µM) and 90 µL of 5 nM fluorescently-tagged high affinity binding peptide FITC-Ahx-AAQLRRIGDKVNLRQKLLN-NH_2. Peptide and protein were dissolved in PBS: 0.05% Tween-20 buffer at pH 7.4. Reagents were incubated for 30 min at room temperature prior to reading. Bubbles were removed and 8 data points were generated. Nonlinear regression was then used to process the data, giving the K_D of the fluorescently-tagged peptide to be 5.2 nM.

To confirm reproducibility of data, a Z-prime test was undertaken, in which 24 wells containing 100 µM mNoxa (Ac-AAQLRRIGDKVNLRQKLLN-NH_2) and 24 wells containing DMSO were screened. The means (µ) and standard deviations (σ) were inputted into the following equation:

$$Z' = 1 - \frac{3(\sigma p + \sigma n)}{|\mu p - \mu n|}$$

The Z-prime produced a value of 0.78, which means that the assay gives excellent reproducibility.

Inhibition screens were carried out using 10 nM Mcl-1, 5 nM fluorescently-tagged peptide and 100 µM of inhibitor. Compounds were dissolved in DMSO. A positive control (mNoxa) and negative control (DMSO) were used to define the minimum and maximum values. Compounds which demonstrated inhibition by fluorescence polarization but also demonstrated excessive fluorescence activity were

excluded. Compounds showing inhibition were then diluted in 10-fold dilutions (from 100 µM to 10 pM well concentration) to generate IC_{50} values, in duplicate. The IC_{50} of mNoxa was calculated to be 0.65 µM and the K_i 0.22 µM. IC_{50} and K_i values were calculated using GraphPad Prism Version 5.0 software.

5. Molecular Docking

The X-ray crystal structure of Mcl-1 with a modified Noxa ligand[2] (PDB ID 2NLA) was utilized for docking calculations. The ligand was removed and the protein was prepared using the Protein Preparation application from the Schrodinger Suite 2015-2.[3] Ligands were generated using Maestro drawing tools and prepared using the LigPrep application from the Schrodinger Suite 2015-2. Binding site grid generation was preformed using the Receptor Grid Generation application from the Schrodinger 2015-2 suite using the bound ligand from PDB entry 2NLA as the base co-ordinates. Docking was performed with protein flexibility using the Induced Fit[4] application from the Schrodinger Suite 2015-2. A total of 100 possible binding confirmations were generated and grouped into clusters using a 1.0 Å root-mean-square tolerance. Confirmations with the lowest glide-emodel score were docked again using Induced Fit to generate the lowest energy confirmation. Figures were generated using the Chimera package from the Computer Graphics Laboratory, University of California, San Francisco (supported by NIH P41 RR-01081).

6. Cell Culture

The MIA PaCa-2 cell line was established from tumor tissue of the pancreas obtained from a 65-year-old Caucasian male, BxPC-3 cell line was derived from a 61 year old female with a primary adenocarcinoma of the pancreas. Cell lines were purchased from the European Collection of Cell Cultures (ECACC, Porton Down, UK). MIA-Pa-Ca-2 cells were cultured in DMEM media with 2 mM L-glutamine, 100 U/mL penicillin, 100 µg/mL streptomycin and 10% FCS. BXPC-3 cells were cultured in RPMI-1640 media containing 2 mM L-glutamine, 100 U/mL penicillin, 100 µg/mL streptomycin and 10% FCS. Cells were sub-cultured twice weekly and maintained at 37 °C and 5% CO_2.

7. MTS Cytotoxicity Assay

The anti-proliferative activity of the compounds studied was assessed by MTS assay (Promega) following the manufacturer's instructions. Cells were seeded at 5 x 10^3 / 100 µL in a 96-well plate and left untreated or treated with DMSO (vehicle control), or compounds **1-4** (100 µM – 10 pm, in well concentration) in triplicate for 72 h at 37 °C with 5% CO_2. Following this, MTS assay reagent was added to each well and the plates incubated for 4 h at 37 °C with 5% CO_2. The absorbance was measured at 492 nm using the BMG Labtech POLARstarOPTIMA microplate reader. IC_{50} values were calculated using GraphPad Prism Version 5.0 software.

8. UV Absorbance Screen

10 µM solutions of each compound in water were analysed using UV absorbance spectroscopy between 200 and 800 nm using a Perkin-Elmer Lamda 25 UV/Vis spectrometer.

[1] B. Zhou, X. Li, Y. Li, Y. Xu, Z. Zhang, M. Zhou, X. Zhang, Z. Liu, J. Zhou, C. Cao, B. Yu, R. Wang, *ChemMedChem* **2011**, *6*, 904-921.

[2] P. E. Czabotar, E. F. Lee, M. F. van Delft, C. L. Day, B. J. Smith, D. C. S. Huang, W. D. Fairlie, M. G. Hinds, P. M. Colman, *Proc. Nat. Acad. Sci.* **2007**, *104*, 6217-6222.

[3] G. M. Sastry, M. Adzhigirey, T. Day, R. Annabhimoju, W. Sherman, *J. Comput.-Aided Mol. Des.* **2013**, *27*, 221-234.
[4] W. Sherman, H. S. Beard, R. Farid, *Chem. Biol. Drug Des.* **2006**, *67*, 83-84.
